# 3D printed Ti6Al4V bone scaffolds with different pore structure effects on bone ingrowth

**DOI:** 10.1186/s13036-021-00255-8

**Published:** 2021-01-21

**Authors:** Fuyuan Deng, Linlin Liu, Zhong Li, Juncai Liu

**Affiliations:** 1 Department of Orthopaedics, The Affiliated Hospital of Southwest Medical University, Luzhou, 646000 Sichuan China; 2 Sichuan Provincial Laboratory of Orthopaedic Engineering, Luzhou, 646000 Sichuan China; 3 School of Mechanical Engineering, Sichuan University, Chengdu, 610065 Sichuan China

**Keywords:** Selective laser melting, Bone scaffold, Pore geometry, Computational fluid dynamics, Bone ingrowth

## Abstract

The microstructure of porous scaffolds plays a vital role in bone regeneration, but its optimal shape is still unclear. In this study, four kinds of porous titanium alloy scaffolds with similar porosities (65%) and pore sizes (650 μm) and different structures were prepared by selective laser melting. Four scaffolds were implanted into the distal femur of rabbits to evaluate bone tissue growth in vivo. Micro-CT and hard tissue section analyses were performed 6 and 12 weeks after the operation to reveal the bone growth of the porous scaffold. The results show that diamond lattice unit (DIA) bone growth is the best of the four topological scaffolds. Through computational fluid dynamics (CFD) analysis, the permeability, velocity and flow trajectory inside the scaffold structure were calculated. The internal fluid velocity difference of the DIA structure is the smallest, and the trajectory of fluid flow inside the scaffold is the longest, which is beneficial for blood vessel growth, nutrient transport and bone formation. In this study, the mechanism of bone growth in different structures was revealed by in vivo experiments combined with CFD, providing a new theoretical basis for the design of bone scaffolds in the future.

## Introduction

In recent years, due to its good biocompatibility and corrosion resistance, titanium and its alloys have been employed for more than 4 decades in clinics [1]. The application of titanium bone scaffolds in bone defect repair has attracted wide attention and is considered a feasible method to repair bone defects beyond the critical defect value. Titanium alloys have a low modulus relative to other alloys, with a Young’s modulus ranging from 7 to 30 GPa [2]. Although the elastic modulus of titanium is still larger than that of human bone, compared with tantalum, stainless steel and other metal materials, the stress masking effect caused by the elastic modulus mismatch is much smaller. The matching degree of titanium implants with the host bone is also higher, and the incidence of bone resorption and implant loosening is also reduced [3]. Porous structures are considered to be an effective method to eliminate elastic modulus mismatches [4–6]. Porous titanium scaffolds with a low elastic modulus can further reduce the stress masking effect, fit well with the host bone, reduce bone absorption, and promote rapid bone formation and integration at the bone-implant interface [7]. Because it is difficult to accurately control the pore size, porosity, pore shape, and pore connectivity with traditional manufacturing techniques, the microstructure of a constructed porous structure scaffold is uncontrollable [8]. In recent years, the rapid development of 3D printing technology, such as selective laser melting (SLM), selective laser sintering, electron beam melting and other technologies, has made it possible to prepare porous structures with controllable microstructures [9–12]. Based on the computer-aided design (CAD) modeling method, CAD technology has been used to create porous microstructure models with controllable porosity and connectivity. The advantage is that the model is relatively simple to build, which is convenient for mechanical analysis, and 3D printing technology can be used to quickly materialize the model. Therefore, research on 3D printed porous bone scaffolds has been substantial in recent years.

Existing research has mainly focused on the design of porous scaffolds with mechanical properties that match the host bone tissue. Additionally, research studies on the effect of the pore size or porosity on the bone ingrowth of different porous structures has been conducted. After the properties are matched with the host bone tissue, the subtle changes in the pore shape have a great influence on the adhesion and proliferation of osteoblasts. The pore shape of the scaffold plays a decisive role in cell growth. Arun et al. [13] conducted an overall study on the stiffness, strength, permeability and stress concentration of six scaffold structures with porosities ranging from 68.46–90.98%. The results show that the pore shape affects the permeability, stiffness, strength and strength of the Ti6Al4V bone scaffold stress concentration factor. The research of Bidan et al. [14] shows that the optimization of the shape of the pore size can increase the growth rate of the bone tissue to the porous scaffold, and the cells grow faster in the square pores. Bael et al. [15] studied the local curvature and pore shape, and the results showed that an obtuse angle was more likely to cause cell blockage than an acute angle. The results of Urda et al. [16] indicate that the straight edges and convexities in the pore structure are the most unfavorable for cell growth. However, these studies on porous scaffold structures are limited to cell experiments, and the growth of cells in vivo is very different from that of a cell culture in vitro. Therefore, it is of certain guiding significance to analyze the osteogenic performance of porous bone scaffold shapes in vivo for the future design of bone tissue scaffolds.

The pore size and porosity also have a great influence on bone ingrowth. Weihu Yang et al. [11] prepared a porous titanium scaffold with a pore diameter of approximately 650 μm by selective laser melting technology, which had better bone ingrowth and better bone integration at the bone-scaffold interface than those with pore sizes of 500 and 900 μm. Xijing He et al. [5] prepared a porous titanium scaffold with a pore diameter of 650 μm by selective laser melting technology, which had a better bone growth effect than those with pore sizes of 400, 500, and 1100 μm. Taniguchi et al. [17] showed that SLM porous titanium scaffolds with a porosity of approximately 65% and a pore diameter of approximately 600 μm could achieve better stability and bone growth than SLM porous Ti6Al4V implants with pore diameters of 300 μm and 900 μm. In fact, a higher porosity and pore size are more conducive to bone ingrowth, although the strength of the scaffold will be reduced [18, 19]. Therefore, when designing scaffolds, we should choose scaffolds with a high porosity and pore size to improve bone growth while ensuring scaffold strength.

In this paper, four common porous structures (DIA, TC, CIR, CU) with a pore size of 650 μm and a porosity of 65% were selected and designed by CAD software. After SLM was used to manufacture the scaffolds, the mechanical experiments showed that the scaffolds had sufficient strength to meet the needs of in vivo experiments. After implantation into the distal femur of 24 rabbits, micro-CT and hard tissue section analyses were performed at 6 and 12 weeks, respectively, to evaluate the bone ingrowth in vivo. A computational fluid dynamics (CFD) method was used to study the relationship between the pore structure parameters and hydrodynamic properties from the perspective of fluid mechanics to reveal the impact of the pore structure on bone formation and scaffold performance. Thus, the optimized porous structure can provide a theoretical scaffold for further research on 3D printed porous bone scaffolds suitable for the human body.

## Materials and methods

### Design and manufacturing of scaffolds

Unigraphics NX (Siemens PLM Software, Germany) was used to design four common porous scaffolds (DIA, TC, CIR, CU) with a porosity of 65% and a pore size of approximately 650 μm. The porosity of the scaffold is the percentage of the structure in a complete solid: 1-V_p_/V_s_.

where V_p_ is the volume of the structural unit and V_s_ is the volume of the complete solid.

**Fig. 1:**
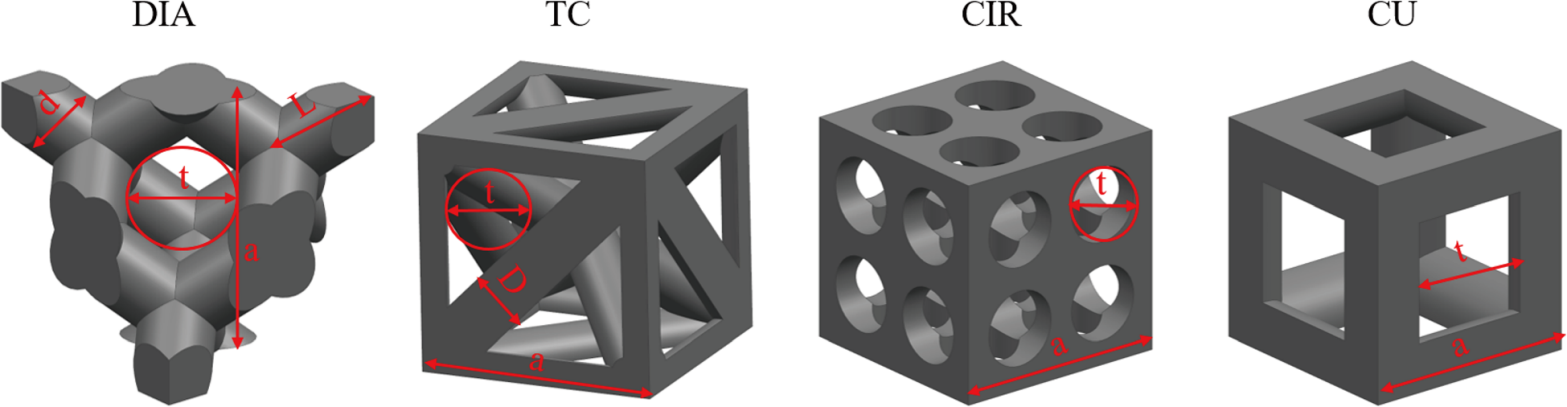
Schematic diagram of the design parameters of the four structures

To obtain structures with the same porosity and pore size, we conducted a parametric design for the design parameters of all structures, as shown in Fig. 1. The pore size of the structure is ‘t’, the unit length is ‘a’, and the strut diameter is ‘D’. The pore size t of the structure was determined by means of the maximum tangential circle. All structures are regular geometric structures, so a mathematical relationship between the design parameters and porosity and pore size can be established. Since the CIR model was not constructed using struts, the pore size t is used to represent its volume.

The volume of the four structures can be expressed by Eq. (1):

1 $$ {V}_{DIA}=4\pi {LD}^2-\frac{585}{1024}{D}^3\tan \left[\frac{1}{2}\operatorname{arccos}\kern0.5em \left(\frac{1}{3}\right)\kern0.5em \right]-\frac{15\sqrt{6}}{128}\pi {D}^3+\frac{135}{2048}{D}^3 $$

$$ {V}_{TC}=\left(3\pi /4\right)\ast {D}^2\mathrm{a}\kern0.5em \left(1+\sqrt{2}\right)\kern0.5em +\kern0.5em \left(2\sqrt{3}\pi {D}^2a/4\right)\hbox{-} \left(3\pi /4\right)\ast {D}^3\left(1+\sqrt{2}\right)-\left(2\sqrt{3\pi }{D}^3/4\right) $$

$$ {V}_{CU}=\frac{3\pi }{4}{D}^2a-\frac{4}{4}\pi {D}^3 $$

$$ {V}_{CIR}={a}_3-\frac{\pi }{4}{t}^2a+\frac{8}{4}\pi {t}^3 $$

The pore size of the four structures can be expressed by Eq. (2):

2 $$ {\mathrm{t}}_{\mathrm{TC}}=\kern0.5em \left(2\hbox{-} \sqrt{2}\right)\mathrm{a}-D $$

$$ {\mathrm{t}}_{DIA}=\frac{\sqrt{6}}{3}\mathrm{a}-D $$

$$ {\mathrm{t}}_{CU}=\mathrm{a}-D $$

In the design, first, the strut diameter was calculated when the cell structure porosity was 65% and then the value of the cell length was calculated when the pore size was 650 μm and the strut diameter D was unchanged. All scaffolds were printed using SLM technology. The 3D printing was carried out by using the FS271M System of Sichuan Farsoon Turing Additive Manufacturing Technology Co., Ltd. The machine was equipped with a 500 W laser with a spot size of 70 μm. The layer thickness and scanning speed were 30 μm and 300 mm/s, respectively. After completing the SLM process, all implants were heated in argon at 800 °C for 2 h and then ultrasonically cleaned with ethanol and distilled water three times (15 min each time).

### Characterization of the scaffolds

The micro-CT technique was used to analyze the structure of the porous titanium scaffold, and then Mimics21.0 (Materialise’s interactive medical image control system, Italy) software was used to reconstruct and measure the scaffold and its surrounding bone tissue in 3D. Parameters such as the pore size of the scaffold, strut size, porosity and surface area were included. The pore size, strut size, porosity and surface area of the scaffold were measured. The data obtained via micro-CT for the measurement of the scaffold parameters are accurate with a small error. Moreover, a large number of studies in the literature have applied micro-CT for the measurement of scaffold parameters, which proves its reliability and accuracy [5, 17, 18, 20, 21].

### Mechanical property of the scaffolds

**Fig. 2:**
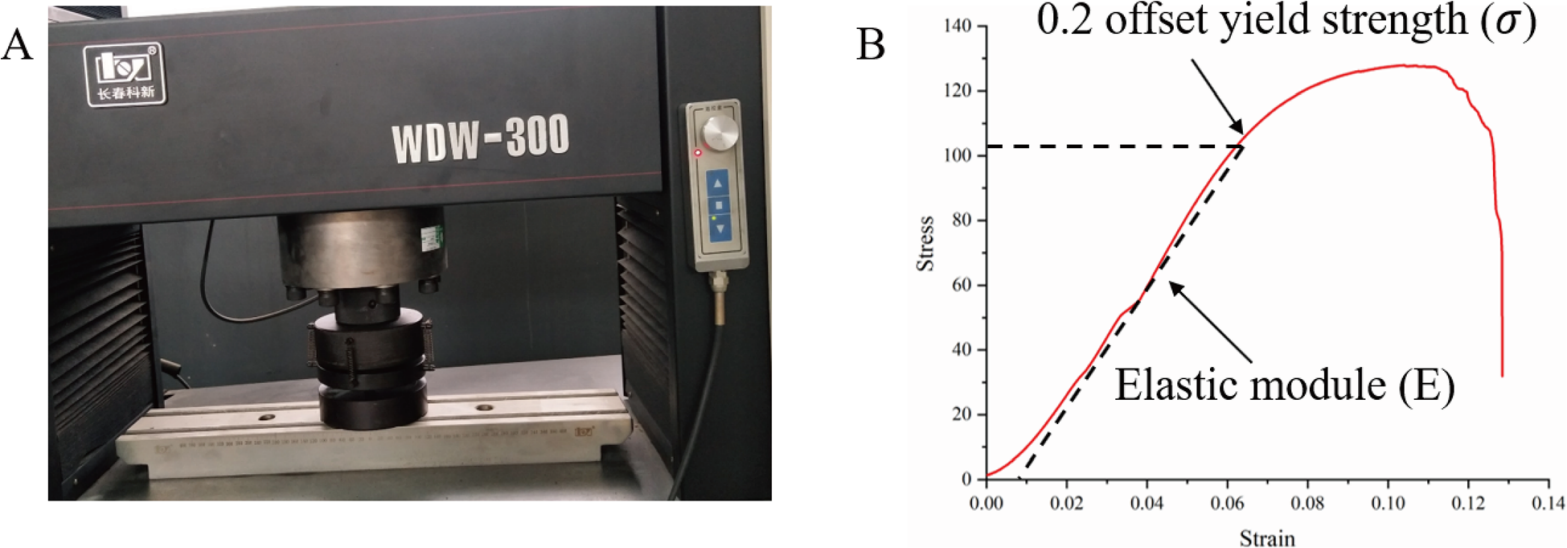
A Material universal testing machine; B DIA stress-strain curve

Mechanical experiments were carried out according to the ISO-13314 standard. A rectangular scaffold (*n* = 5, 10 × 10 × 12 mm) was used for the compressive strength experiments. A material universal testing machine (WDW-300; Changchun Kexin Testing Instrument Co., Ltd.) was used for the mechanical experiments, as shown in Fig. 2a. During the experiment, compression was performed at a speed of 1 mm/min. The elastic modulus (E) and compressive strength (*σ*) of the porous scaffold were obtained from the stress-strain curve of the material. The elastic modulus of the scaffold was calculated according to the maximum slope in the elastic region of the stress-strain curve. The compressive strength of the scaffold was calculated by the 0.2% offset method, as shown in Fig. 2b, which is the typical stress-strain curve of the DIA.

### Surgical procedure

**Fig. 3:**
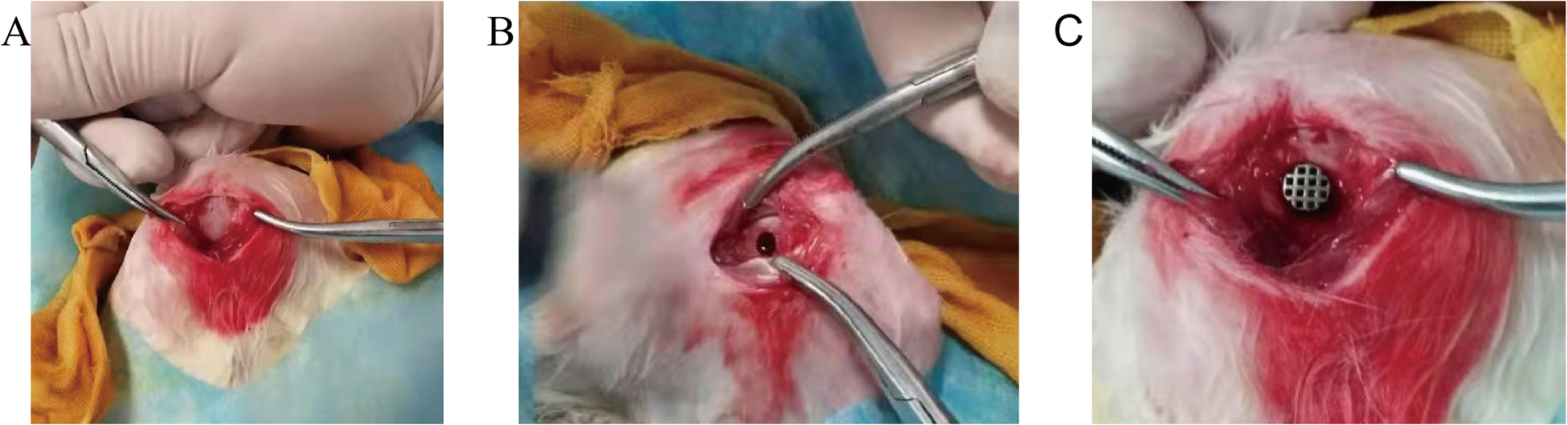
**a** Exposure of the distal lateral condyle of the femur; **b** A defect (5 mm in diameter and 8 mm in depth) was drilled from the lateral femoral condyle of the rabbit at low speed; **c** Titanium scaffold implanted into the bone defect

In this study, a total of 24 adult New Zealand white rabbits (2.5–3.0 kg) from the Experimental Animal Center of Southwest Medical University were selected. There were 12 females and 12 males. The temperature of the breeding room is 24 °C, and the humidity is 60%. The rabbits have free access to water and food. The experimental plan was approved by the Southwest Medical University Laboratory Animal Protection and Welfare Committee in accordance with international standards. Twenty-four rabbits were randomly divided into two groups according to the implantation time (6 weeks, 12 weeks). In each group, 12 rabbits with a total of 24 femurs were implanted with four different cylindrical scaffolds (*n* = 6, φ5 × 8 mm). Intravenous injection of 3% pentobarbital sodium (30 mg/kg) under general anesthesia and 0.5% lidocaine local anesthesia. After skin preparation disinfection, a 3 cm longitudinal incision was made in both femoral condyles for surgery. The skin and subcutaneous tissue were cut to separate the muscles, and the periosteum was cut to expose the femoral condyle (Fig. 3a). A hole with a diameter of 5 mm and a depth of 8 mm was drilled in sequence on the lateral side of the femoral condyle with a low-speed drill (Fig. 3b). When drilling holes, physiological saline was used to reduce the temperature to prevent tissue necrosis caused by local high temperatures. After the drilling was completed, the scaffold was implanted (Fig. 3c), and the wound was sutured in turn. Three days after surgery, intramuscular injection of cephalosporin antibiotics was used for anti-infective treatment. At 6 weeks and 12 weeks after the operation, the rabbits were sacrificed by intravenous air injection. The femurs were removed, washed with formalin-fixed water, dehydrated with ethanol, infiltrated and embedded, and the hard tissue was sectioned into 200 μm sections using an EXAKT E300CP hard tissue slicer. After grinding and polishing, slices of approximately 70 μm were made. The slices were stained by the HE staining method and observed under an optical microscope. We observed whether there was new bone tissue, microvascular tissue or fibrous tissue inside the scaffold.

### Micro-CT analysis

To assess the effectiveness of new bone formation, the rabbit femurs implanted with porous scaffolds were scanned using micro-CT (Micro-CT100, SCANCO Medical AG). The scanning parameters were set as follows: X-ray source voltage = 90 kV; beam current = 200 μA; scanning resolution = 17.2 μm. After scanning the sample, the projection is reconstructed and segmented into a binary image and further analyzed with Mimics 21.0 (Materialise, Belgium). The internal space of the scaffold and bone tissue growth into the scaffold were defined as the volume of interest, and the bone volume (BV) and total pore volume (TV) were measured by micro-CT for further detailed data analysis. By calculating the ratio of BV to TV (BV/TV), the BT/TV value with higher bone growth performance was quantitatively evaluated, indicating that more bone had grown into the scaffold.

### Histological evaluation

The fixed femoral condyle was dehydrated in ethanol and then embedded with methyl methacrylate, and a saw blade was used to cut a 50 μm thick section along the long axis of the cylindrical scaffold. After staining with 1.2% trinitrophenol and 1% acid magenta (Van-Gieson staining), the images were observed under a light microscope via fluorescence microscopy.

### CFD simulation

The permeability, velocity and internal velocity streamlines of the different scaffolds were evaluated by CFD. Due to the symmetry of the structure, only 2 × 2 × 2 units were used for analysis to save calculation time. Assuming that the fluid is incompressible, the Navier Stokes equation [22] was adopted for calculation:

3 $$ \rho \frac{\theta \mathrm{v}}{\theta \mathrm{t}}-u{\nabla}^2V+\rho \left(v\cdot \nabla \right)v+\nabla p=F,\nabla .V=0 $$

where ρ, v, and μ represent the fluid density (kg/m^3^), velocity of fluid flow (m/s) and dynamic fluid viscosity (kg/m/s), respectively. ∇ is the del operator, and p and F represent the pressure (MPa) and force (N), respectively.

The permeability K of the four structures was calculated by Darcy’s law equation [23]:

4 $$ K=\frac{v.u.L}{\Delta P} $$

where v, L, and ∆P represent the inlet fluid flow velocity (m/s), model length (m), and pressure difference (MPa), respectively.

**Fig. 4:**
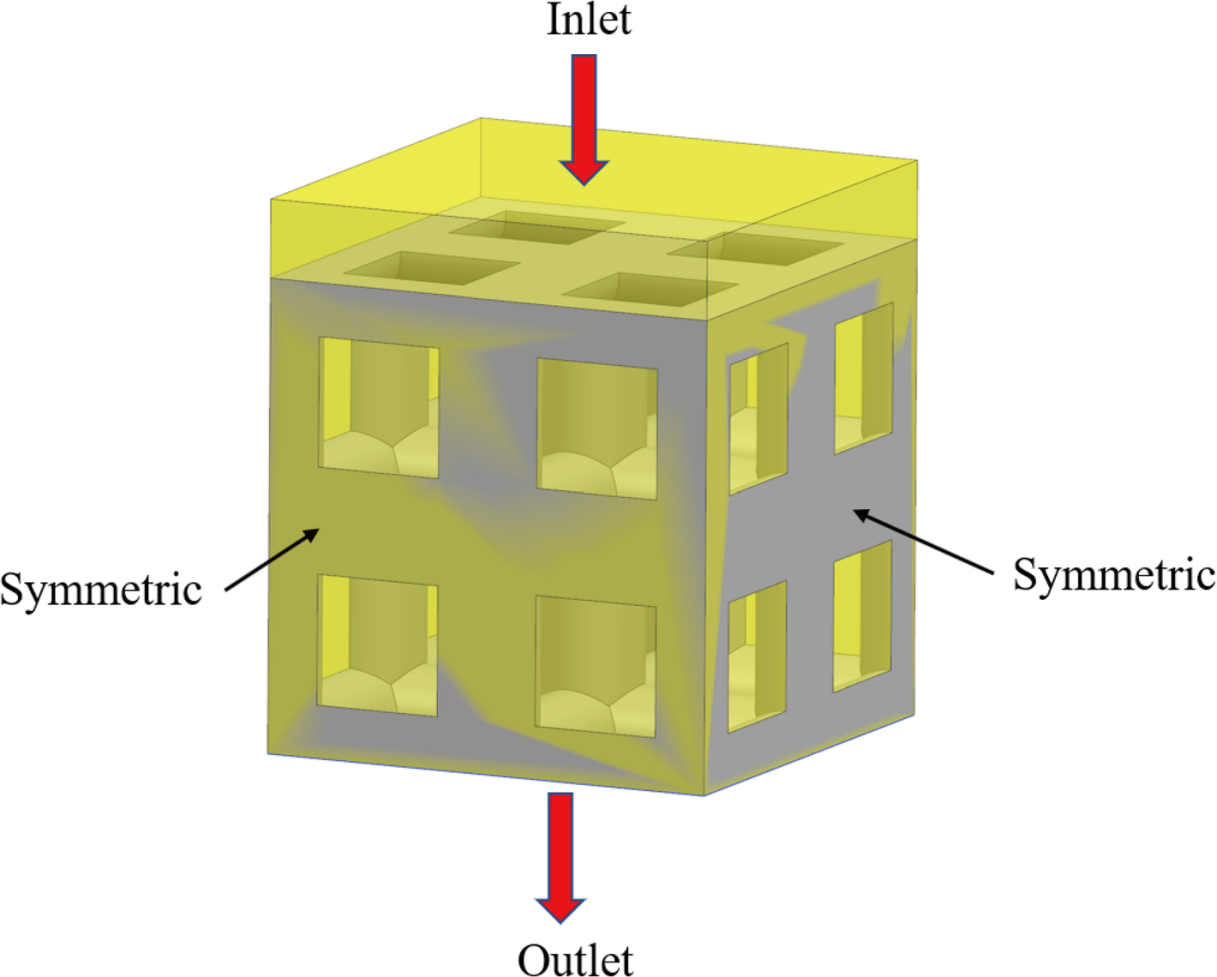
Schematic diagram of the CFD simulation boundary conditions

Using Ansys Fluent software, the CFD simulation model was analyzed and calculated, as shown in Fig. 4. During the calculation, the inlet velocity was set at 0.1 mm/s, the outlet pressure was set at 0, the fluid density was set at 1050 kg/m^3^, and the viscosity was set at 0.0037 kg/m/s [24]. The extra fluid domain above the structure was used to avoid boundary effects.

### Statistical analysis

The analysis was performed using SPSS software (SPSS Inc., Chicago, Il, USA). All the data are expressed as the mean ± standard deviation and were analyzed with one-way ANOVA. In all cases, the results are considered statistically significant with a *p*-value less than 0.05.

## Results and discussion

### Characterization of the porous titanium scaffolds

**Fig. 5:**
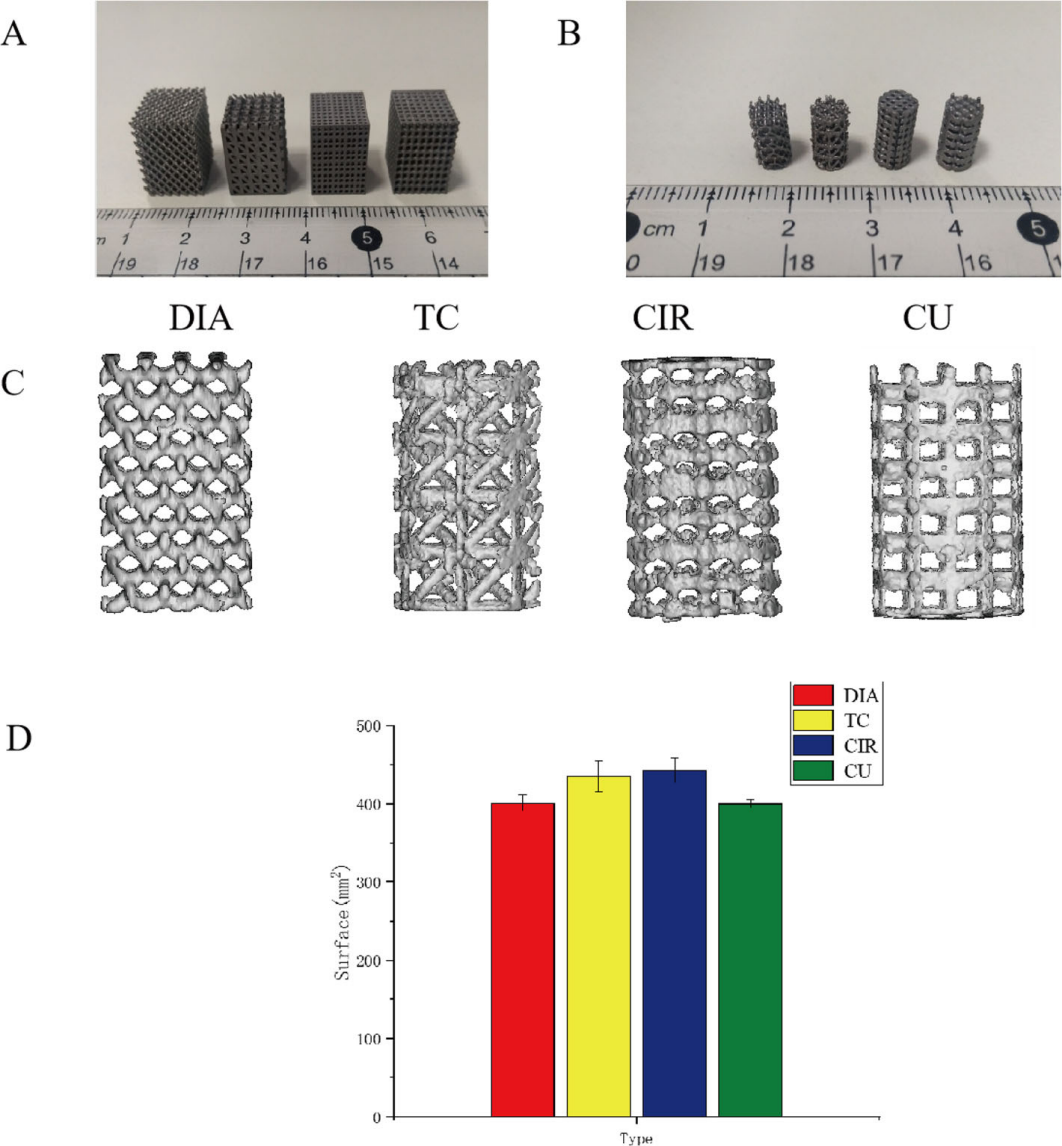
**a** Photo of a 3D printed titanium scaffold for the mechanical experiments; **b** Photo of the four cylindrical scaffolds for the in vivo experiments; **c** Micro-CT reconstruction of the porous titanium scaffold; **d** Surface area of four kinds of porous titanium scaffolds measured after 3D reconstruction

Figure 5a is an enlarged photo of the 3D printed titanium scaffold manufactured by SLM for the mechanical experiments. Figure 5b is an enlarged photo of the four cylindrical titanium scaffolds manufactured by SLM. Figure 5c is an image obtained after the 3D reconstruction of the titanium scaffold. The printed scaffold has good consistency with the 3D reconstruction model, and the aperture and scaffold size are well controlled and uniform. Figure 5d shows the measured scaffold surface area after the 3D reconstruction of the four porous titanium scaffolds.

In this experiment, the bone scaffold was fabricated using SLM technology [15, 20], through which complex 3D metal parts can be manufactured with good controllability and repeatability. Therefore, the printed entity conforms to the CAD model with minimal error, which is helpful for controlling the parameters of the porous structure and reducing the experimental error. At the same time, relevant studies have shown that porous Ti6Al4V scaffolds have good biocompatibility and are conducive to cell adhesion and proliferation [25], which also ensures the safety of our porous titanium scaffolds implanted in animals. Table 1 shows the theoretical values of the structural parameters of the porous titanium scaffolds with four different topological structures and the actual values of the measured parameters after 3D reconstruction, including the porosity, pore size, strut size and volume. The difference between the theoretical and actual structural parameters is small, which means that the printed scaffold is of high quality. Small values indicate that the quality of the printed scaffold is high.

**Table 1:** Structural parameters of the porous titanium scaffolds with four different topological structures

Sample	Porosity (%)		Pore size (um)		Unit size (mm)		Volume (mm^3^)	
	T	A	T	A	T	A	T	A
DIA	64.5	64.8 ± 1.2	650	650 ± 2.9	1.6	1.6 ± 0.3	55.9	55.4 ± 0.4
TC	65	65.3 ± 1.1	650	648 ± 4.1	1.86	1.84 ± 0.4	54.8	55 ± 0.4
CIR	64.5	64.8 ± 1.2	680	678 ± 4.8	2	2.0 ± 0.1	55.7	56 ± 1.2
CU	65	65 ± 1.1	660	663 ± 5.9	1.2	1.23 ± 0.1	55	56 ± 0.6

*A* Actual value; *T* Theoretical value

### Mechanical properties of the scaffold

**Fig. 6:**
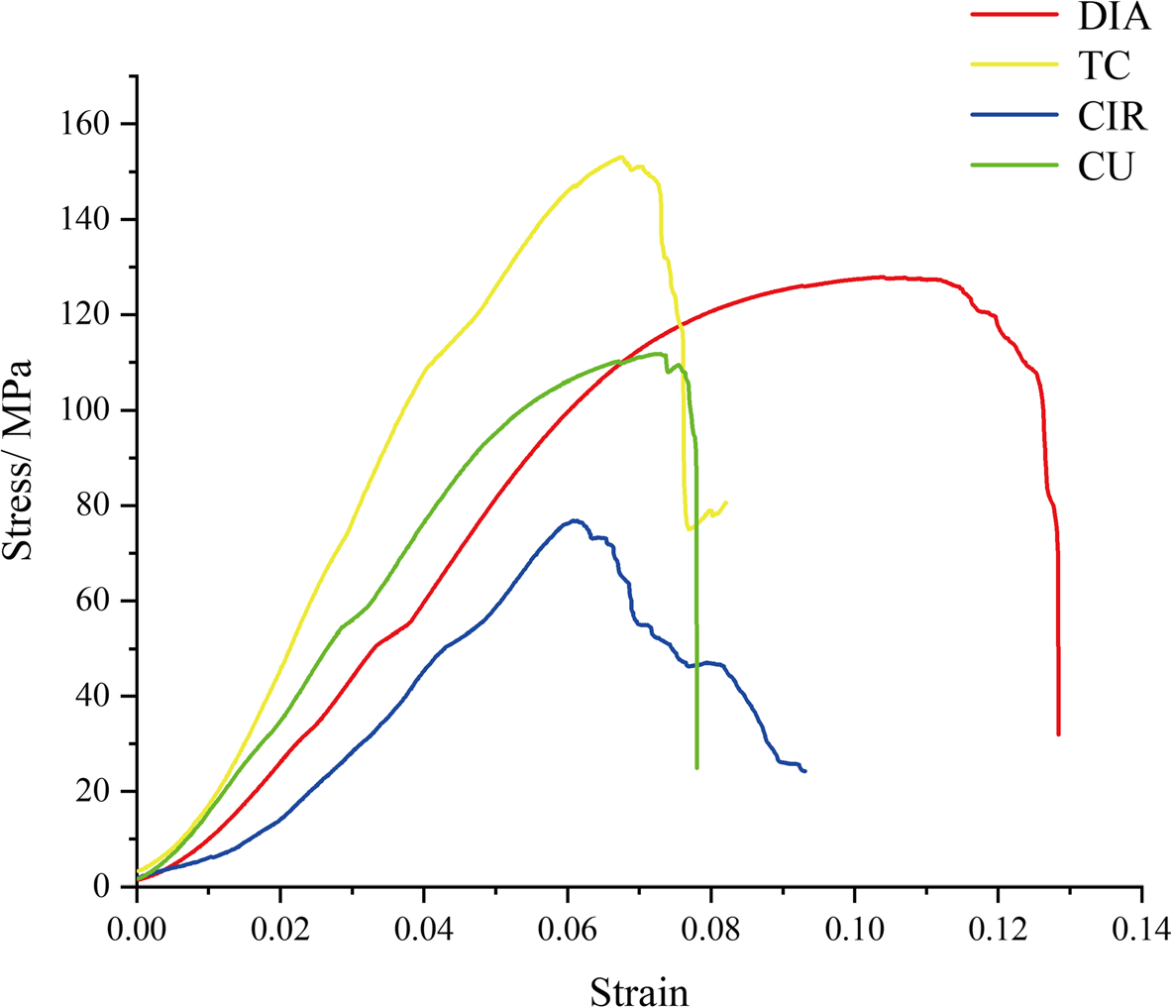
Stress-strain curves of the four kinds of scaffold structures

Figure 6 shows the stress-strain curves of the four scaffolds. It can be seen from the figure that the scaffold with the CIR structure is the lowest (49 MPa), which is caused by the fact that the strut is not homogeneous, and the weakest in the middle part. The yield strengths of the other three strut types are relatively close. Additionally, the elastic modulus and yield strength values of the four strut structures measured by the experiment are shown in Table 2. Studies have shown that the elastic modulus of bone trabeculae ranges from 0.1–4.5 GPa [26], and the yield strength of the proximal tibia and proximal femur ranges from 0.56–55.3 In this paper, except for the CIR structure, the yield strengths of all the other three structures exceed this range. The elastic modulus of the four structures range from 1.9–4.2 GPa, which also well matches the elastic modulus of the host bone tissue. Therefore, the structure in this paper can not only build an elastic modulus that matches the host bone tissue but also ensure that the scaffold has a high yield strength, which can be well applied in bone tissue scaffolds.

**Table 2:** Elastic modulus and yield strength of the four scaffold structures

Scaffold type	Elastic modulus(E/GPa)	Yield strength(*σ* /MPa)
DIA	2.1 ± 0.8	106 ± 6
TC	4.4 ± 0.3	107 ± 3
CIR	1.8 ± 0.5	49 ± 2
CU	2.6 ± 0.1	96 ± 5

### Micro-CT analysis

**Fig. 7:**
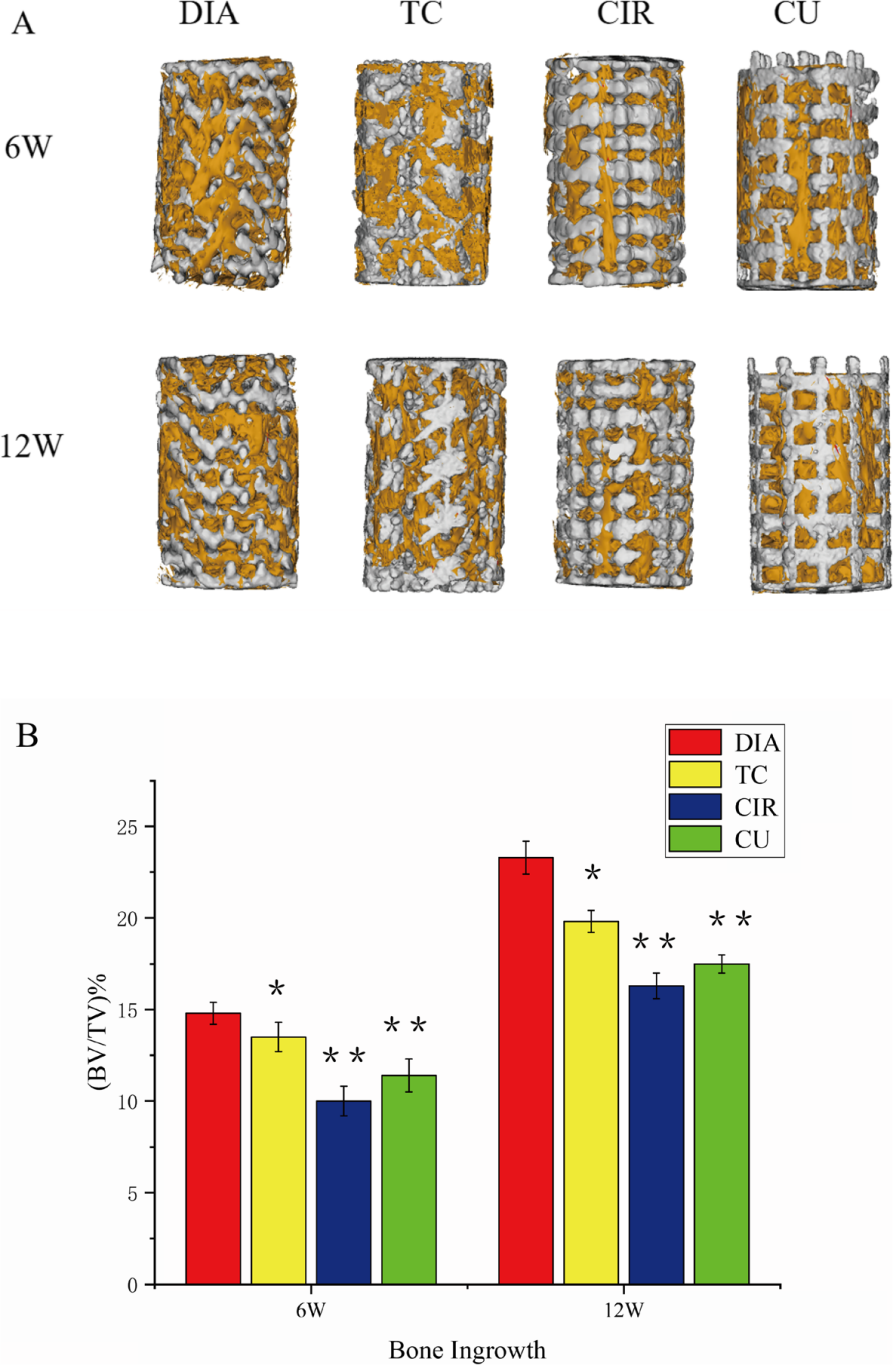
**a** Micro-CT reconstruction of the distal femur of rabbits after 6 and 12 weeks of titanium scaffold implantation. White represents the bone scaffold, and yellow represents new bone. **b** The titanium scaffold was implanted in the distal femur of rabbits. *P < 0.05, **P < 0.001 compared with DIA

The titanium scaffold was implanted at the distal end of the rabbit femur, and the bone scaffold was removed for micro-CT measurements after 6 and 12 weeks. The formation of bone in the scaffold was evaluated by this technique. Figure 7a shows the 3D reconstruction image of the scaffold and new bone. As seen from the reconstructed images, the bone tissue in the scaffold gradually increased with increasing time. At 6 and 12 weeks, the DIA, CU, TC, CIR and CIR models were in sequence from high to low, which was consistent with the ratio between BV and TV (BV/TV) calculated by the quantitative analysis in Fig. 7b. Within 6 and 12 weeks, the BV/TV ratios were 15.2 and 23.1% for DIA, 13.7 and 19.1% for CU, 11.4 and 18.3% for TC, and 9.8 and 16.9% for CIR, respectively. Since we accurately controlled the porosity and pore size of the porous scaffolds, the most likely reason for this osteogenic difference was the pore shape. Bael [15] found that according to in vitro experimental results, circular pore structures are more prone to pore blockage than non-circular pore structures. This structural change may influence the delivery of nutrients and oxygen inside the scaffold and thus affect bone growth. The pore shape may be one of the reasons that the CIR structure has the least new bone. The DIA structure consists of 16 struts of equal length, each with an angle of 109.5°, which is similar to the intertrabecular angle of human cancellous bone measured by Natalie [27]. Therefore, this trabecular bone-like structure may be beneficial for bone growth. As is known, the bone scaffold porosity and void size have an important effect on cell adhesion, proliferation, differentiation and new bone formation.

### Histological analysis

**Fig. 8:**
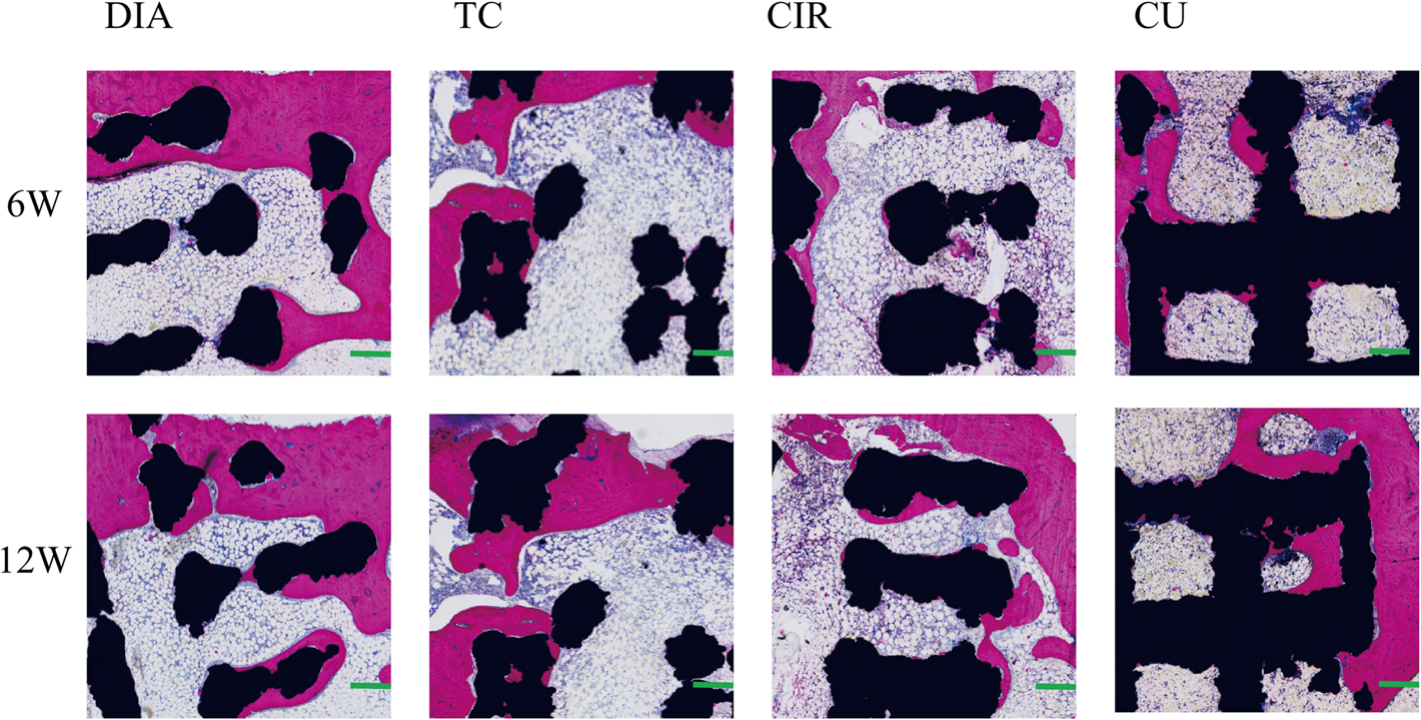
Histological sections of dehydrated embedded samples of the bone scaffolds obtained at 6 weeks and 12 weeks were stained. Red represents the bone tissue, and black represents the scaffold. Original magnification: 10.0; scale bar: 1 mm

**Fig. 9:**
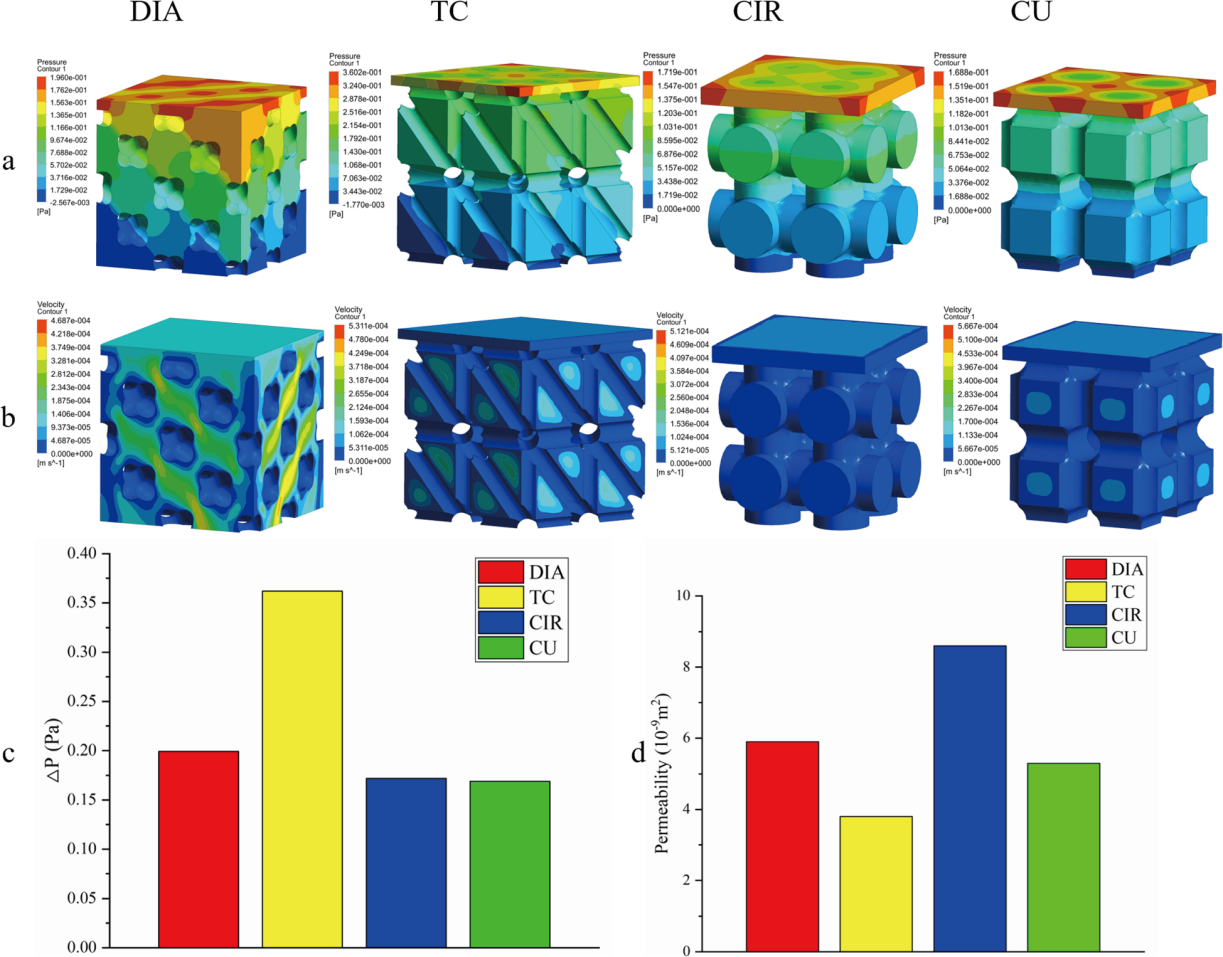
Fluid simulation results of the four structures

**Fig. 10:**
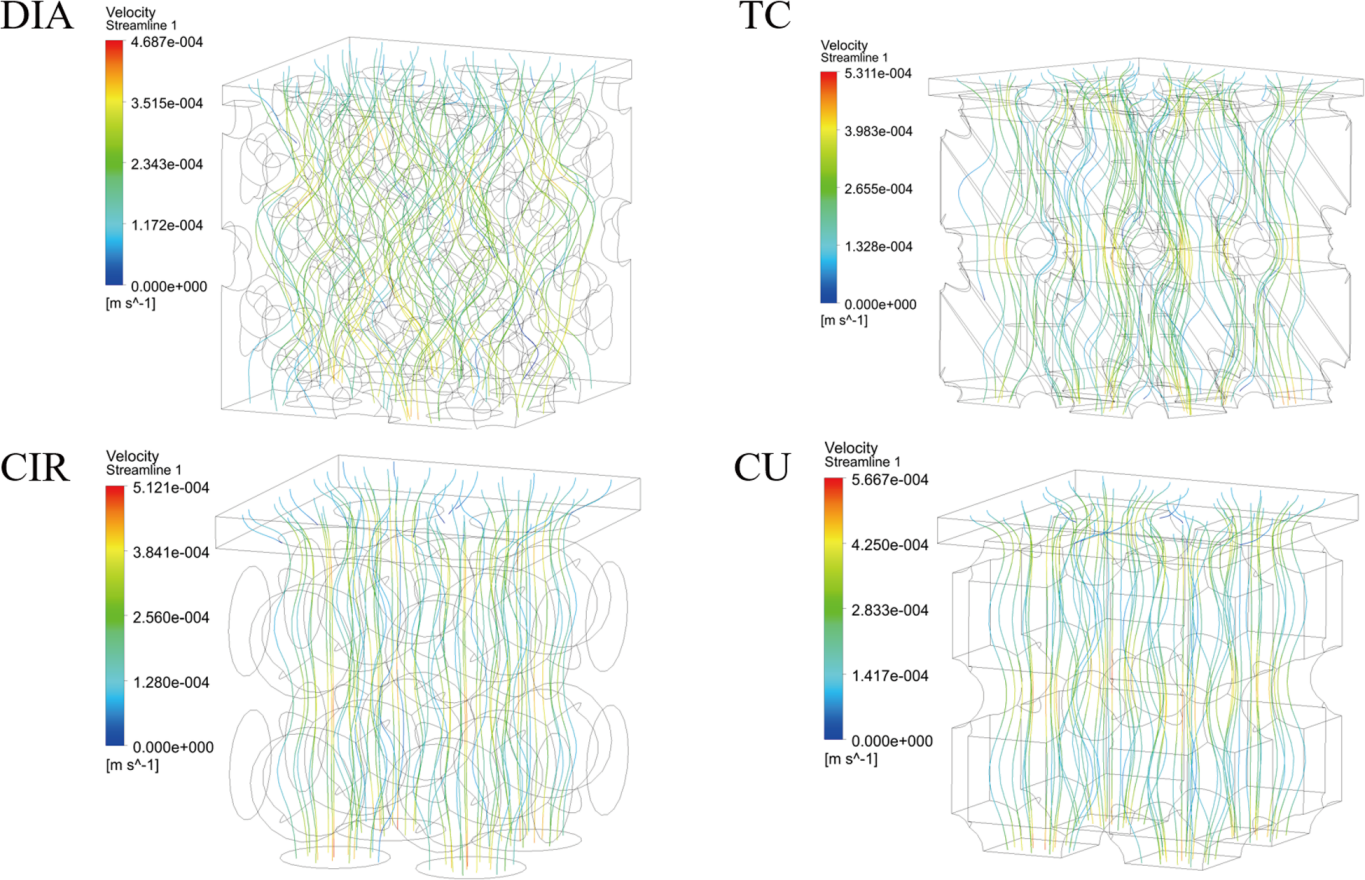
Velocity flow diagrams for the four structures

The animals were sacrificed in batches at 6 and 12 weeks, and the samples harvested from the animals were fixed in 10% formalin, dehydrated with a series of ethanol solutions (70, 80, 90, 95 and 100% X2) and subsequently embedded in poly (methylmethacrylate) (PMMA, Cool-Set-A, Aorigin, Chengdu, China). Sections (10–20 μm) were made with a diamond histological saw (SAT-001, Origin, Chengdu, China) and stained with methylene blue (Sigma)/basic fuchsin (Sigma) for histological observation. Bone growth was qualitatively analyzed through the bone growth in the section diagram, and the histological section diagram of the scaffold obtained after methylene blue (Sigma)/basic fuchsin (Sigma) staining is shown in Fig. 8. These section diagrams clearly show the formation of bone tissue in the scaffold, and with increasing time, the amount of bone tissue in the pores of the scaffold increased gradually. The maximum amount of the new bone mass was exhibited in the DIA structure, and the minimum amount was in the CIR structure. This result is consistent with the qualitative and quantitative results obtained by micro-CT in Fig. 9 and Fig. 10. Therefore, according to the results of the slices, we further verified the accuracy of the experimental results.

### CFD analysis

The permeability and velocity of fluid flow in the internal structure of the scaffold have a great influence on the growth of bone tissue in the scaffold [28]. The fluid flow inside the scaffold can provide necessary oxygen and nutrients for the growth of cells. Excessive permeability is not conducive to the adhesion of cells on the surface of the scaffold, while too low of a permeability cannot provide sufficient nutrients [29].

Figure 9a shows the pressure cloud diagram of the scaffold. The pressure gradually decreases from the highest point of the inlet to the lowest point of the outlet. For different structures, the pressure is uniformly distributed within the scaffold. Figure 9b shows the velocity cloud map of the scaffold. For the CIR and CU structures, the internal structure is relatively simple, so the velocity difference in the scaffold is not very large. For the DIA and TC structures, due to their complex internal structure, there is also a large velocity difference [30]. The DIA velocity cloud shows that the velocity on the side of the scaffold is significantly greater than the fluid flow inside the scaffold. This result indicates that the DIA structure can accelerate fluid flow inside the scaffold structure, facilitating fluid flow to more areas of the scaffold. Figure 9c shows the pressure difference △P at the inlet and outlet of the four structures. It can be seen from the figure that the pressure drop difference in the DIA, CIR and CU structures is not very great, while the pressure difference in the TC structure is more than twice as large. This finding indicates that the internal obstruction of the TC structure has a large effect on the fluid, which does not facilitate the flow of fluid inside the structure or through more areas inside the structure. Figure 9d shows the permeability of the four structures, which is calculated according to Eq. 4. The permeability range of the proximal tibia is 0.467 × 10^− 9^ m^2^ to 14.810^− 9^ m^2^ [31]. The permeability range of the four structures in this paper is 3.8 × 10^− 9^ m^2^ to 5.9 × 10^− 9^ m^2^. Therefore, the permeability is in line with the requirements of bone tissue implantation.

Figure 10 shows the velocity flow diagrams for the four scaffold structures. It can be seen from the figure that the velocity streamlines of the TC, CIR and CU structures are relatively simple and flow through fewer areas inside the structure. Compared to the other three structures, the DIA structure has a low internal flow rate, and the lower speed allows cells to attach more easily to the surface of the scaffold, thus promoting bone growth in the body. The speed of the other three structures at the intersection of the struts is significantly higher than that at the pores, which can promote the migration of cells to a deeper depth and make the cells less likely to adhere to the surface of the struts. Therefore, the cells grew more in the DIA structure than in the other three structures.

The pore structure is an important parameter that affects bone scaffolds and plays an important role in the mechanical properties of cell migration and adhesion tissue formation and nutrient diffusion. There are many in vitro experiments on the pore structure with different conclusions, and many studies have been limited to cell experiments [20, 32–34]. Our previous work [35] confirmed that we can print accurate titanium scaffolds using SLM. It has been confirmed by cell experiments in vivo that titanium scaffolds have good biocompatibility, which has also been confirmed in a large number of studies and can meet the normal adhesion and differentiation requirements of cells. A cell culture is static, while the human environment is dynamic. Thus, cell tests do not completely simulate bone growth in humans. The size of bone defects, blood flow, local inflammatory response, osteocytes involved in bone formation, osteoblasts and various endogenous growth factors released by them, and mechanical stimulations all have an impact on bone growth. Therefore, in vivo experiments are needed for further confirmation. In this study, four kinds of porous titanium alloy scaffolds with different structures made by SLM were selected, and four kinds of scaffolds were implanted at the distal end of rabbit femur to evaluate the growth of bone tissue in vivo to explore the optimal pore structure of bone growth. Based on CFD, the permeability, velocity and flow trajectory of the scaffold structure were calculated. The combination of in vivo experiments and CFD simulations reveals the causes of bone ingrowth in different structures, which provides a new theoretical basis for the design of bone scaffolds in the future.

Before the four scaffolds were implanted in vivo, mechanical experiments were conducted on these structures to measure their elastic modulus and yield strength values. According to the related literature, the elastic modulus of trabecular bone is in the range of 0.1–4.5 GPa, and the yield strength of the vertebrae, proximal tibia and proximal femur ranges from 0.56–55.3 MPa. The results showed that the elastic modulus of the four kinds of scaffolds was within the normal range, which could avoid the stress masking effect caused by a mismatch of the elastic modulus. The yield strength of the four kinds of scaffolds met the strength requirements of orthopedic implants.

Through quantitative and qualitative analysis of the new bone mass, we concluded that structural bone growth in the DIA structure was the best and bone growth in the CIR was the worst, which was further verified in the hard tissue sections. Compared with the results of the in vitro experiments conducted by Bael, circular pore structures are more prone to pore blockage than non-circular pore structures. One of the reasons for this behavior is the decrease in nutrients and oxygen transport inside the scaffold, which affects bone growth. The second reason is that the fluid mechanics shows that the DIA structure can accelerate fluid flow inside the scaffold structure, which is beneficial for the fluid flow to more areas of the scaffold. At the same time, the flow velocity inside the DIA structure is not large. The lower speed can better promote cell adhesion to the surface of the scaffold. Moreover, only when the cells stick together can they proliferate further, thus facilitating bone growth in the body. However, the results of Bael et al. showed that obtuse angles were more likely to cause cell clogging than acute angles, and, in this paper, the TC structure was second only to the DIA structure. The DIA struts have an angle of 109° between them. According to its structural analysis, the DIA structure should grow into a poor structure, but in fact, the opposite is true. We believe that Bael’s research on the bone scaffold angle is limited to the 2D level, while the DIA angle is measured in 3D, so this theory is not completely applicable to the DIA structure. At the same time, according to fluid mechanics analysis, the pressure difference between the inlet and the outlet of the TC structure is more than twice that of the DIA, CIR and CU structures. This result indicates that the internal obstruction of the TC structure is greater, which is not conducive to the flow of fluid inside the structure or through more areas inside the structure, so the delivery of oxygen and nutrients will be relatively less. However, the internal structure of TC is more complex than that of CIR and CU, and the site of internal cell attachment is more than that of the latter two. At the same time, it can be seen that the velocity streamlines of the TC, CIR and CU structures are relatively close. Triangular holes seem to be more conducive to cell proliferation and differentiation than the square and round holes, so the TC structure is better than the CIR and CU structures in terms of bone growth. In summary, based on the in vitro and in vivo experiments, we conclude that the DIA model demonstrated the best structural bone growth.

At present, there are many topological structures used in scaffold design. In this paper, four common structures are selected, and a comparison of the results obtained is limited to these four structures. In addition, the simulation of blood flow by using computer fluid mechanics cannot completely replace an in vivo situation, and the data may be biased to some extent, so further accuracy is needed in future studies.

## Conclusion

Via selective laser melting method, the structures of four different kinds of porous titanium alloy scaffolds with a similar porosity (65%) and aperture size (650 μm) were prepared and investigated through in vivo experiments along with CFD analysis. Combined with existing research studies, the different structures of the bone ingrowth of bone scaffolds were analyzed and the following conclusions were drawn:

1. SLM printing can print high-strength and low-modulus bone scaffolds, with good application prospects in orthopedics.
2. The pore structure has a great influence on bone growth. Among the four different pore structures, the DIA structure demonstrated the best bone growth effect.
3. CFD analysis was performed, and the permeability, flow rate and flow trajectory of the scaffold structure were calculated. The results showed that the internal fluid velocity difference in the DIA structure was the smallest and the fluid flow trajectory was the longest in the scaffold, which was conducive to bone growth.
4. This paper provides a new method for the research of porous scaffolds by combining computational fluid dynamics analysis and in vivo experiments and provides a new basis for the design of future scaffolds.

## Data Availability

Not applicable.

## Abbreviations

DIA: Diamond lattice unit
CFD: Computational fluid dynamics
SLM: Selective laser melting

## References

[1] Zehao J,Jing Z,Zhang T,Xiu P,Cai H, Wei Q, Fan D , Lin X,Song C, Liu Z. Functionalization of 3D-printed titanium alloy orthopedic implants: a literature review[J]. Biomedical Materials. 2020;15(5). 10.1088/1748-605X/ab9078 32369792

[2] Chen Q, Thouas GA (2015). Metallic implant biomaterials. Mat Sci Engineering R-Reports.

[3] Frost HM (2004). A 2003 update of bone physiology and Wolff's law for clinicians. Angle Orthod.

[4] Li L, Shi J, Zhang K, Yang L, Yu F, Zhu L, Liang H, Wang X, Jiang Q (2019). Early osteointegration evaluation of porous Ti6Al4V scaffolds designed based on triply periodic minimal surface models. J Orthopaedic Transl.

[5] Ouyang P, Dong H, He X, Cai X, Wang Y, Li J, Li H, Jin Z. Hydromechanical mechanism behind the effect of pore size of porous titanium scaffolds on osteoblast response and bone ingrowth. Mater Des. 2019;183.

[6] Maietta S, Gloria A, Improta G, Richetta M, De Santis R, Martorelli M. A further analysis on Ti6Al4V lattice structures manufactured by selective laser melting. J Healthcare Engineering. 2019;2019. 10.1155/2019/3212594 PMC6778933 31662833

[7] Li J, Jansen JA, Walboomers XF, van den Beucken JJJP. Mechanical aspects of dental implants and osseointegration: a narrative review. J Mech Behav Biomed Mater. 2020;103. 10.1016/j.jmbbm.2019.103574 32090904

[8] Chen Z, Yan X, Yin S, Liu L, Liu X, Zhao G, Ma W, Qi W, Ren Z, Liao H, Liu M, Cai D, Fang H. Influence of the pore size and porosity of selective laser melted Ti6Al4V ELI porous scaffold on cell proliferation, osteogenesis and bone ingrowth. Mat Sci Engineering C-Materials for Biological Applications. 2020;106. 10.1016/j.msec.2019.110289 31753386

[9] Revilla-Leon M, Meyer MJ, Ozcan M (2019). Metal additive manufacturing technologies: literature review of current status and prosthodontic applications. Int J Comput Dent.

[10] Onal E, Frith JE, Jurg M, Wu X, Molotnikov A. Mechanical properties and in vitro behavior of additively manufactured and functionally graded Ti6Al4V porous scaffolds. Metals. 2018;8(4).

[11] Ran Q, Yang W, Hu Y, She X, Yu Y, Xiang Y, Cai K (2018). Osteogenesis of 3D printed porous Ti6Al4V implants with different pore sizes. J Mech Behav Biomed Mater.

[12] Weissmann V, Bader R, Hansmann H, Laufer N (2016). Influence of the structural orientation on the mechanical properties of selective laser melted Ti6Al4V open-porous scaffolds. Mater Des.

[13] Arjunan A, Demetriou M, Baroutaji A, Wang C. Mechanical performance of highly permeable laser melted Ti6Al4V bone scaffolds. J Mech Behav Biomed Mater. 2020;102. 10.1016/j.jmbbm.2019.103517 31877520

[14] Bidan CM, Kommareddy KP, Rumpler M, Kollmannsberger P, Fratzl P, Dunlop JWC (2013). Geometry as a factor for tissue growth: towards shape optimization of tissue engineering scaffolds. Advanc Healthcare Mat.

[15] Van Bael S, Chai YC, Truscello S, Moesen M, Kerckhofs G, Van Oosterwyck H, Kruth IP, Schrooten J (2012). The effect of pore geometry on the in vitro biological behavior of human periosteum-derived cells seeded on selective laser-melted Ti6Al4V bone scaffolds. Acta Biomater.

[16] Rudrich U, Lasgorceix M, Champion E, Pascaud-Mathieu P, Damia C, Chartier T, Brie J, Magnaudeix A (2019). Pre-osteoblast cell colonization of porous silicon substituted hydroxyapatite bioceramics: influence of microporosity and macropore design. Mat Sci Engineering C-Materials for Biological Applications.

[17] Taniguchi N, Fujibayashi S, Takemoto M, Sasaki K, Otsuki B, Nakamura T, Matsushita T, Kokubo T, Matsuda S (2016). Effect of pore size on bone ingrowth into porous titanium implants fabricated by additive manufacturing: an in vivo experiment. Mat Sci Engineering C-Materials for Biological Applications.

[18] Otsuki B, Takemoto M, Fujibayashi S, Neo M, Kokubo T, Nakamura T (2006). Pore throat size and connectivity determine bone and tissue ingrowth into porous implants: three-dimensional micro-CT based structural analyses of porous bioactive titanium implants. Biomaterials.

[19] Karageorgiou V, Kaplan D (2005). Porosity of 3D biomaterial scaffolds and osteogenesis. Biomaterials.

[20] Zhang B, Pei X, Zhou C, Fan Y, Jiang Q, Ronca A, D'Amora U, Chen Y, Li H, Sun Y, Zhang X (2018). The biomimetic design and 3D printing of customized mechanical properties porous Ti6Al4V scaffold for load-bearing bone reconstruction. Mater Des.

[21] Akiyama H, Morishima T, Takemoto M, Yamamoto K, Otsuka H, Iwase T, Kabata T, Soeda T, Kawanabe K, Sato K, Nakamura T (2011). A novel technique for impaction bone grafting in acetabular reconstruction of revision total hip arthroplasty using an ex vivo compaction device. J Orthop Sci.

[22] Vossenberg P, Higuera GA, van Straten G, van Blitterswijk CA, van Boxtel AJB (2009). Darcian permeability constant as indicator for shear stresses in regular scaffold systems for tissue engineering. Biomech Model Mechanobiol.

[23] Gomez S, Vlad MD, Lopez J, Fernandez E (2016). Design and properties of 3D scaffolds for bone tissue engineering. Acta Biomater.

[24] Sinha R, Le Gac S, Verdonschot N, van den Berg A, Koopman B, Rouwkema J. Endothelial cell alignment as a result of anisotropic strain and flow induced shear stress combinations. Sci Rep. 2016;6. 10.1038/srep29510 PMC4941569 27404382

[25] Li Y, Yang C, Zhao H, Qu S, Li X, Li Y (2014). New developments of Ti-based alloys for biomedical applications. Materials.

[26] Morgan EF, Bayraktar HH, Keaveny TM (2003). Trabecular bone modulus-density relationships depend on anatomic site. J Biomech.

[27] Reznikov N, Chase H, Ben Zvi Y, Tarle V, Singer M, Brumfeld V, Shahar R, Weiner S (2016). Inter-trabecular angle: a parameter of trabecular bone architecture in the human proximal femur that reveals underlying topological motifs. Acta Biomater.

[28] Ali D (2019). Effect of scaffold architecture on cell seeding efficiency: a discrete phase model CFD analysis. Comput Biol Med.

[29] Melchels FPW, Tonnarelli B, Olivares AL, Martin I, Lacroix D, Feijen J, Wendt DJ, Grijpma DW (2011). The influence of the scaffold design on the distribution of adhering cells after perfusion cell seeding. Biomaterials.

[30] Ma S, Tang Q, Feng Q, Song J, Han X, Guo F (2019). Mechanical behaviours and mass transport properties of bone-mimicking scaffolds consisted of gyroid structures manufactured using selective laser melting. J Mech Behav Biomed Mater.

[31] Beaudoin AJ, Mihalko WM, Krause WR (1991). Finite element modelling of polymethylmethacrylate flow through cancellous bone. J Biomech.

[32] Bouet G, Marchat D, Cruel M, Malaval L, Vico L (2015). In vitro three-dimensional bone tissue models: from cells to controlled and dynamic environment. Tissue Engineering Part B-Reviews.

[33] Graziano A, D'Aquino R, Angelis MGC-D, De Francesco F, Giordano A, Laino G, Piattelli A, Traini T, De Rosa A, Papaccio G (2008). Scaffold's surface geometry significantly affects human stem cell bone tissue engineering. J Cell Physiol.

[34] Knychala J, Bouropoulos N, Catt CJ, Katsamenis OL, Please CP, Sengers BG (2013). Pore geometry regulates early stage human bone marrow cell tissue formation and organisation. Ann Biomed Eng.

[35] Wang S, Liu L, Li K, Zhu L, Chen J, Hao Y. Pore functionally graded Ti6Al4V scaffolds for bone tissue engineering application. Mater Des. 2019;168.

